# Gut microbiota and osteonecrosis: A Mendelian randomization study

**DOI:** 10.1097/MD.0000000000041703

**Published:** 2025-03-07

**Authors:** Yong Cai, Chaoqing Zhou, Junjie Guan, Bo Dai, Xingshi Zhang, Jizhao Jiang, Jun Zhao

**Affiliations:** a Department of Orthopaedics, Xishui County People’s Hospital, Across from Hongding Community, Zunyi City, Guizhou Province, China; b Department of Orthopaedics, Zhuhai Hospital of Integrated Traditional Chinese and Western Medicine, Zhuhai, Guangdong, China.

**Keywords:** causal effect, gut microbiota, Mendelian randomization, osteonecrosis, risk factor

## Abstract

Emerging evidence indicates an association between the gut microbiota and the incidence of osteonecrosis (ON), yet the literature has not adequately addressed whether this relationship is causal. This study uses data from the MiBioGen Consortium and the UK Biobank for Mendelian randomization (MR) analysis to identify pathogenic gut microbial taxa associated with ON. Sensitivity analyses confirmed causal relationships, while reverse MR ruled out reverse causation. SNP annotation located genetic variants linked to gut microbiota instrumental variables in ON. The inverse variance weighted method revealed 5 microbial taxa with a causal association with ON, including the order Erysipelotrichales (OR = 2.24, 95% CI = 1.16–4.32, *P* = .02), genus Christensenellaceae R (OR = 0.41, 95% CI = 0.19–0.87, *P* = .02), family Erysipelotrichaceae (OR = 2.24, 95% CI = 1.16–4.32, *P* = .02), family Family XIII (OR = 0.45, 95% CI = 0.21–0.95, *P* = .04), and class Erysipelotrichia (OR = 2.24, 95% CI = 1.16–4.32, *P* = .02). Sensitivity analyses mitigated concerns regarding heterogeneity, directional pleiotropy, and outliers (*P* > .05). However, the reverse MR showed no causal effect of ON on these taxa. SNP (single-nucleotide polymorphism) annotation pinpointed 20 host genes associated with ON pathogenesis. These findings lay the groundwork for microbiota-targeted therapies and deepen our understanding of the gut-bone axis in osteonecrosis.

## 1. Introduction

Osteonecrosis (ON), also known as avascular necrosis, is a debilitating pathological condition characterized by the death of bone tissue due to inadequate blood supply.^[1]^ Furthermore, treating ON remains challenging, with limited therapeutic options available.^[2]^ ON affects various skeletal sites, including the hip, knee, shoulder, mandible, and ankle, and is associated with significant pain, functional impairment, and a reduced quality of life.^[3]^ The prevalence of ON varies widely, with rates ranging from 3% to 20% depending on etiology, such as corticosteroid use, alcohol abuse, or systemic diseases like systemic lupus erythematosus.^[2]^ Despite extensive research, the underlying mechanisms of ON remain poorly understood, with current studies emphasizing multifactorial interactions, including genetic predispositions, lipid metabolism disorders, and inflammatory responses.^[4,5]^ However, these factors alone do not fully explain the ON ‘s occurrence in all cases, suggesting that additional mechanisms, such as genetic predisposition, may be involved and require further exploration.

In recent years, research within the medical domain has increasingly focused on the gut microbiota – a diverse and complex assembly of microorganisms, including bacteria, fungi, and viruses, hat inhabit the human gastrointestinal tract.^[6]^ Disruption of the balance of gut microbiota, caused by various factors, can lead to a range of adverse health outcomes such as metabolic disorders, obesity, diabetes, and even cancer.^[7]^ Recent study has suggested a potential link between the gut microbiota and bone health. The gut microbiota has been shown to influence bone remodeling through various mechanisms, including modulation of immune responses, production of short-chain fatty acids, and regulation of vitamin D metabolism.^[8]^ Dysbiosis, characterized by an imbalance in gut microbial composition, has been associated with several bone-related conditions, including rheumatoid arthritis and osteoporosis.^[9]^ Despite these findings, the causal role of gut microbiota in osteonecrosis remains unclear, necessitating further investigation.

This study hypothesizes that specific gut microbiota taxa have a causal relationship with the risk of osteonecrosis. By leveraging Mendelian randomization (MR), we aim to identify gut microbial taxa with significant causal effects on ON and elucidate potential biological pathways involved. To address these gaps, our study utilizes MR analysis, which leverages genetic variation as an instrumental variable (IV) to explore causal relationships while minimizing confounding factors and reverse causation. This approach enables the identification of pathogenic gut microbial taxa potentially involved in ON pathogenesis and advances our understanding of the gut-bone axis.

## 2. Materials and methods

### 2.1. Data sources

The GWAS (Genome Wide Association Studies) summary statistics for gut microbiota, which served as the exposure variable, were sourced from the MiBioGen consortium(https://mibiogen.gcc.rug.nl/). In this study, the gut microbiota was classified into 211 taxa, comprising131 genera, 35 families, 20 orders, 16 classes, and 9 phyla. The analysis included a comprehensive set of 5717,754 SNPs. The GWAS data for ON, derived from the International Classification of Diseases 9th and 10th editions, consisted of 970 cases (including idiopathic aseptic necrosis cases, instances of drug-induced osteonecrosis, and other forms) and 294,793 healthy controls of European ancestry. This data was obtained from the FinnGen Project Database (https://r7.finngen.fi/; 7th data release). Key attributes, such as effect size, effect allele, and *P*-value for each SNP were retained for further examination. In our study, we finally included 196 taxa after excluding unknown gut microbes (Table S1, Supplemental Digital Content, http://links.lww.com/MD/O450).

### 2.2. Genetic instrument selection

**Figure 1.**
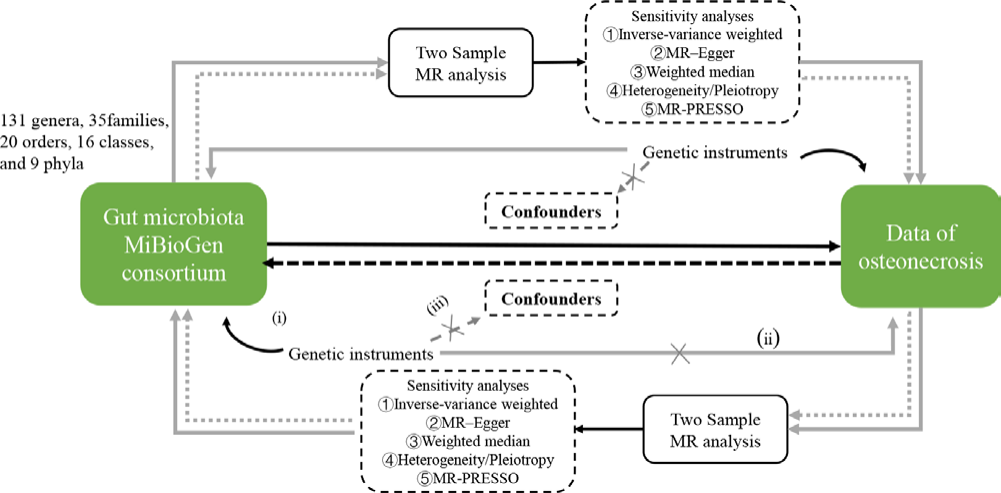
Workflow of present study and basic assumptions of MR analysis. Genetic instruments must be (A) associated with the exposure; (B) independent of confounders; (C) independent of the outcome. MR = mendelian randomization, MR-PRESSO = Mendelian randomization pleiotropy residual sum and outlier.

The *P*-value threshold for SNP selection was set at *P* < 1×10^−5^to ensure sufficient coverage of IVs while maintaining statistical power. A stricter threshold (*P* < 5×10^−8^) was deemed too restrictive for this analysis, as it might exclude relevant SNPs, increasing the risk of weak instrument bias.^[10]^ To ensure independence, SNPs with *r*^2^≥0.001 or physical distance≤10,000 kb were excluded.^[11]^ Confounding factors were minimized by leveraging the MR framework, which uses genetic variants as IVs under the assumption of random assortment. Pleiotropy and heterogeneity were evaluated using MR-Egger regression, weighted median estimation, and MR-pleiotropy residual sum and outlier (MR-PRESSO) tests. Sensitivity analyses, including leave-one-out analyses and Cochran *Q* test, further ensured robustness by identifying potential biases from individual SNPs or horizontal pleiotropy.^[10]^ Outlier SNPs were identified and removed using the MR-PRESSO test. In the reverse MR analysis, the significance threshold for ON was set to *P* < 5×10^−6^ in order to expand the statistical effect. Furthermore, we calculated the *F*-statistic to evaluate the strength of the SNPs, and *F* ≥ 10 was considered a sufficient threshold to control for weak IV bias^[12]^ (as shown in Fig 1).

### 2.3. Estimation of causal effect and sensitivity analysis

We identified potential reverse causal associations between SNPs linked to the gut microbiota and ON through the implementation of the MR Steiger Filtering Test.^[13]^ The sensitivity analyses conducted in this study encompassed various tests, including MR-PRESSO, heterogeneity, multiple validity, and leave-one-out tests. MR-PRESSO was employed to identify and remove outliers from further analysis.^[14]^ The heterogeneity of IVs was assessed using Cochran *Q* test, with a significance level of *P* < .05 indicating the presence of heterogeneity, requiring an explanation for its source. Pleiotropy was evaluated using the MR-Egger intercept test, where a *P*-value >.05 indicated the absence of pleiotropy. Lastly, the leave-one-out method was utilized to assess the influence of each individual SNPs on the analysis results by removing 1 SNP at a time.^[14]^ The results of these analyses were visualized using scatterplots, funnel plots, and forest plots, respectively.

### 2.4. SNP annotation

In order to identify SNP-associated motifs, we utilized web-based tools (https://biit.cs.ut.ee/gprofiler/snpense) for SNP annotation. g: SNPense serves as an instrumental resource for associating human SNP identifiers with their correlated genes, and offering predicted variant effects. By entering SNP data on the designated website page and initiating conversion, the corresponding gene information is revealed.^[15]^ This tool maps human SNP rs codes to gene names, providing chromosomal coordinates and predicted variant effects.^[15]^

### 2.5. Statistical analysis

All relevant statistical analyses in the paper were conducted using the TwoSampleMR and MRPRESSO packages^[16]^ in R language (version 4.3.1).

## 3. Results

### 3.1. Overview

After conducting a thorough screening for SNPs associated with the exposure and eliminating linkage disequilibrium, we found that all 1555 remaining SNPs, classified into 16 classes, 20 orders, 32 families, and 54 genera, exhibited *F*-statistics >10 (Table S2, Supplemental Digital Content, http://links.lww.com/MD/O450). Using the IVW approach, we detected a significant causal association between the gut microbiota and the risk of ON. Ultimately, we identified 5 microbial taxa, including 1 order, 1 genus, 2 families, and 1 class, that demonstrated a significant association with a *P*-value of <.05.

### 3.2. Causal effect of gut microbiota on ON

**Figure 2.**
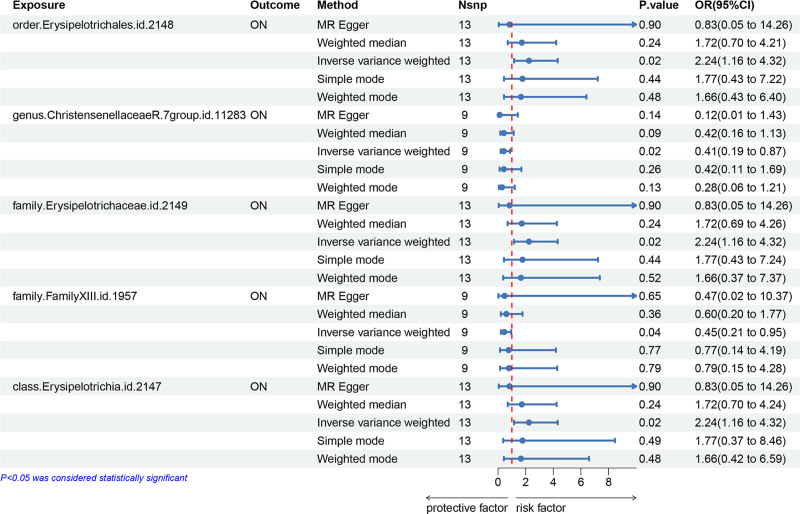
The positive results of IVW analysis in MR analysis. This forest plot shows the odds ratios for microbial taxa significantly associated with ON risk. Taxa such as Erysipelotrichales demonstrated strong positive associations (OR = 2.24, *P* = .02), suggesting a potential role in ON pathogenesis. IVW = inverse variance weighted, MR = Mendelian randomization, ON = osteonecrosis, OR = odds ratio, SNP = single-nucleotide polymorphism.

The results of the IVW analysis showed that the order Erysipelotrichales, class Erysipelotrichia, and family Erysipelotrichaceae were positively correlated with risk of ON. Conversely, higher abundance of genus ChristensenellaceaeR and family FamilyXIII were associated with lower risk of ON. Specifically, using MR Egger method, we observed an inverse association between the phylum Proteobacteria and a positive association between the family Defluviitaleaceae with the risk of ON. These findings highlight the clinical significance of microbial abundance changes. The strong positive association of Erysipelotrichales with ON risk suggests that dysbiosis involving the taxa may exacerbate ON development. Conversely, the protective role of Christensenellaceae underscores its potential as a target for microbiota-based interventions aimed at reducing ON risk. Moreover, employing the Weighted median method, we identified a positive association between the order Victivallales and the class Lentisphaeriawith the risk of ON (Table S3, Supplemental Digital Content, http://links.lww.com/MD/O450). The positive results of IVW analysis in MR analysis were shown in Figure 2.

**Figure 3.**
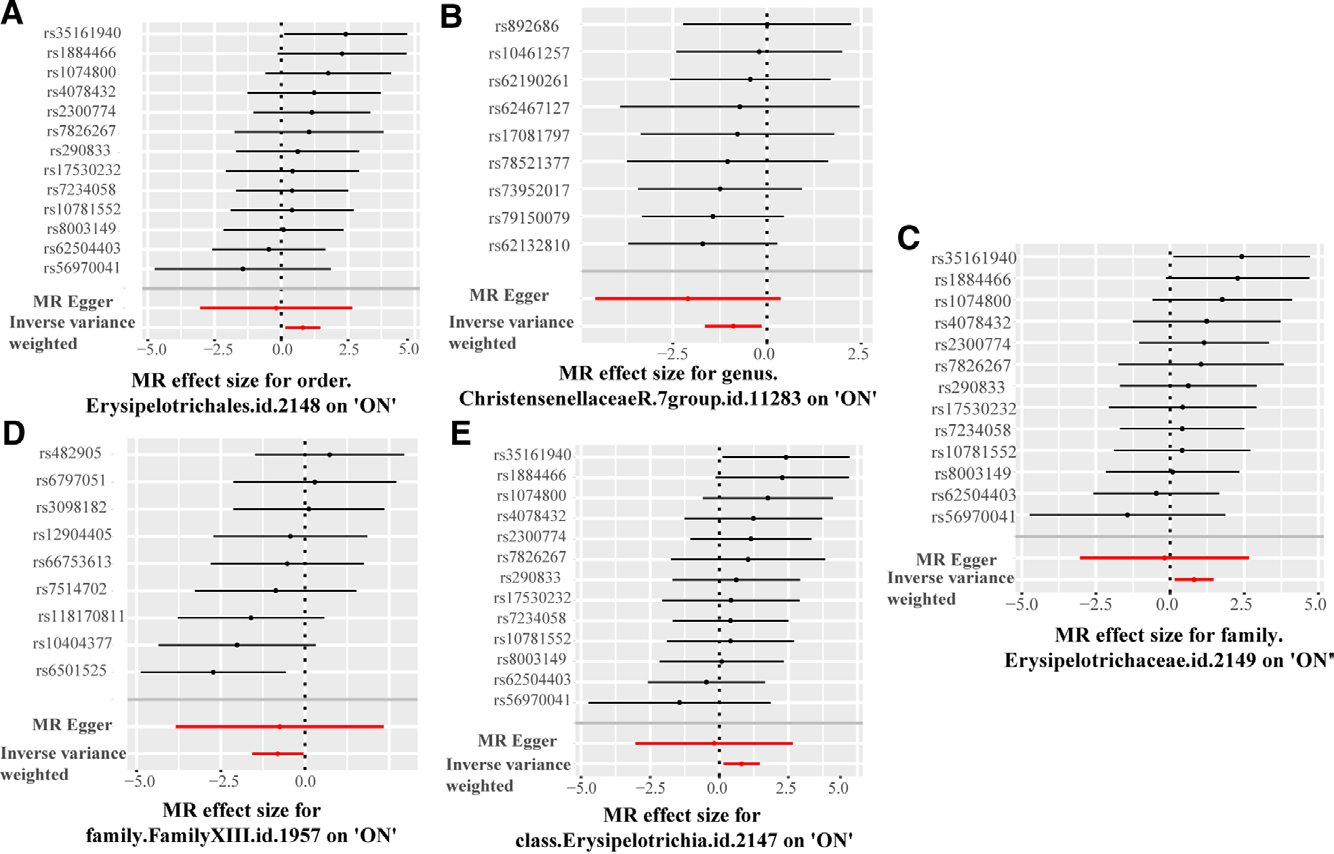
Causal association between causal microbial taxa with ON. (A) Forest plot for order.Erysipelotrichales.id.2148; (B) forest plot for genus.ChristensenellaceaeR.7group.id.11283; (C) forest plot for family.Erysipelotrichaceae.id.2149; (D) forest plot for family.FamilyXIII.id.1957; (E) forest plot for class.Erysipelotrichia.id.2147. ON = osteonecrosis, SNP = single-nucleotide polymorphism; the results of the forest plot.

**Figure 4.**
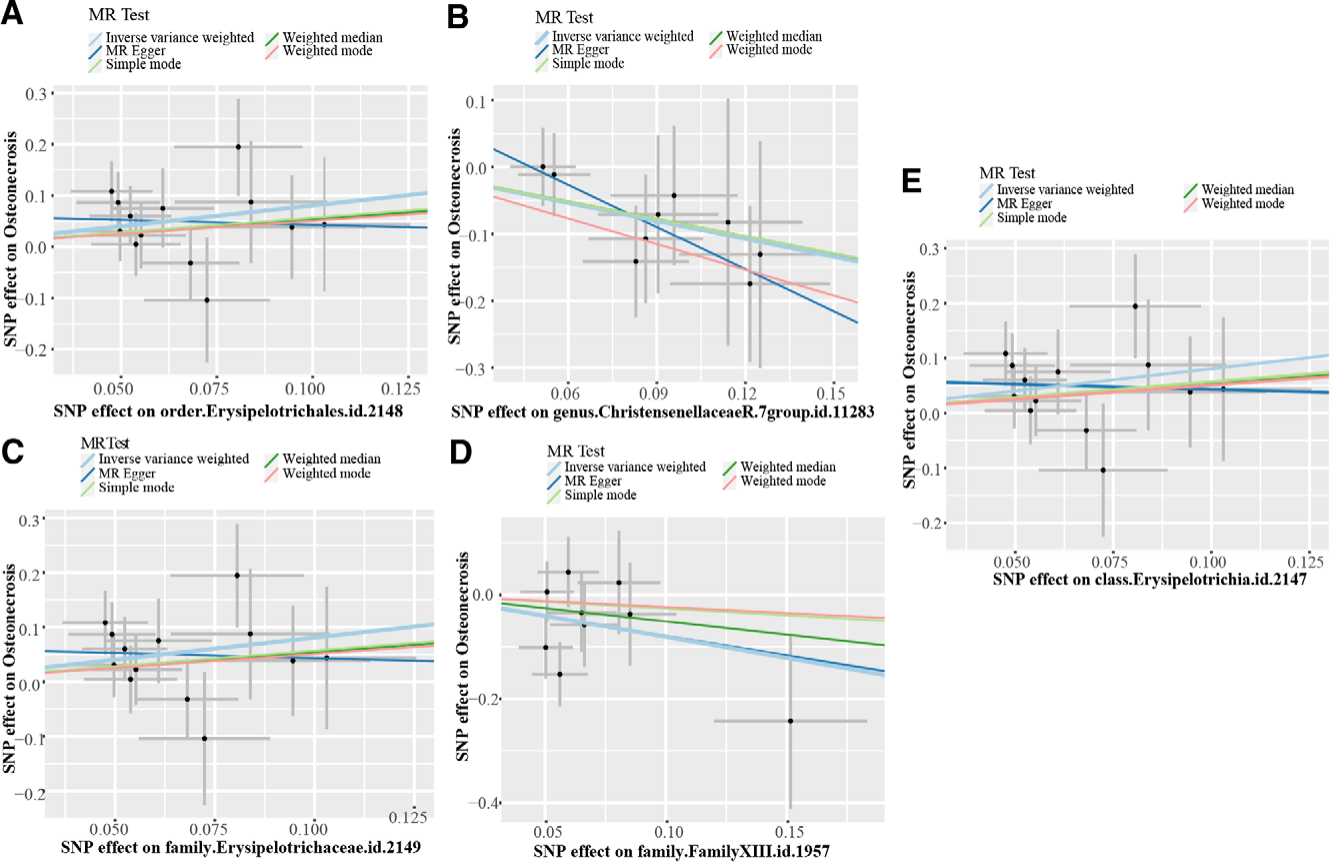
Causal association between causal microbial taxa with ON. (A) Scatter plot for order.Erysipelotrichales.id.2148; (B) scatter plot for genus.ChristensenellaceaeR.7group.id.11283; (C) scatter plot for family.Erysipelotrichaceae.id.2149; (D) scatter plot for family.FamilyXIII.id.1957; (E) scatter plot for class.Erysipelotrichia.id.2147. ON = osteonecrosis, SNP = single-nucleotide polymorphism; light blue lines represent estimations with IVW method.

The MR Steiger test did not identify a negative causal relationship between bacterial taxa and ON. In order to mitigate potential bias effects, the Egger Intercept test did not detect significant horizontal pleiotropy (*P* > .05, Table S4, Supplemental Digital Content, http://links.lww.com/MD/O450). Furthermore, MR-PRESSO did not identify outlier SNPs, and Cochrane *Q* test did not detect heterogeneity (*P* > .05, Table S5, Supplemental Digital Content, http://links.lww.com/MD/O450). Leave-one-out analyses showed that no single SNP unduly influenced the results (Fig. S1, Supplemental Digital Content, http://links.lww.com/MD/O449). The results were demonstrated on the forest plot and scatter plot in Figures 3 and 4.

### 3.3. Reverse MR analysis

**Figure 5.**
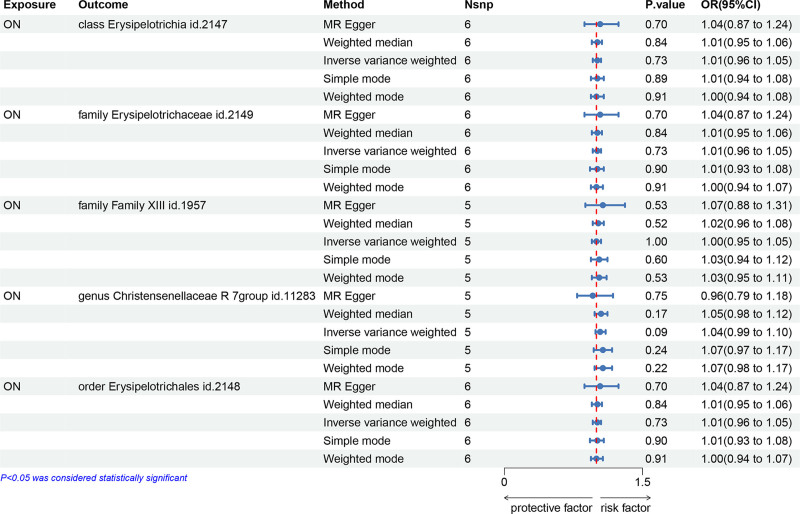
The results of the reverse MR analysis. MR = Mendelian randomization, OR = odds ratio, ON = osteonecrosis, SNP = single-nucleotide polymorphism.

To ascertain whether ON contributed as a pathogenic risk factor to various instances of previously mentioned bacterial taxa, a reverse MR analysis was executed across 5 critical bacterial taxa. The outcomes derived from the IVW, MR-Egger, and Weighted Median approaches indicated no significant correlation between ON and any of the bacterial taxa under consideration (*P* > .05, refer to Table S6, Supplemental Digital Content, http://links.lww.com/MD/O450). Additionally, both the MR-Egger and MR-PRESSO tests yielded no signs of pleiotropy, while the Cochrane *Q* test confirmed the absence of heterogeneity (*P* > .05), as presented in Tables S7 and S8, Supplemental Digital Content (http://links.lww.com/MD/O450). Thus, we deduce that there was no reverse causality between ON and these said bacterial taxa. The outcomes of the reverse MR analysis were visually depicted in Figure 5.

### 3.4. SNP annotation

Subsequently, we annotated the pertinent SNPs (Table S9, Supplemental Digital Content, http://links.lww.com/MD/O450) and the findings were detailed in Table 1 provided. Ultimately, we identified 20 host genes that potentially correlate with pathogenic gut microbiota in ON patients.

**Table 1:** SNP annotation of intestinal flora IVs.

id	chr	Start	End	Strand	Gene ids	Gene names
rs10461257	4	155,209,852	1.55E + 08	+	ENSG00000185149, ENSG00000250910	NPY2R, MAP9-AS1
rs17081797	18	69,888,324	69,888,324	+	ENSG00000150637	CD226
rs62132810		−1	−1			–
rs62190261		−1	−1			–
rs62467127	7	118,456,871	1.18E + 08	+	ENSG00000106013	ANKRD7
rs73952017	18	1,779,608	1,779,608	+	ENSG00000266450, ENSG00000266602	–
rs78521377	10	124,759,805	1.25E + 08	+	ENSG00000203791, ENSG00000258539	EEF1AKMT2
rs79150079	5	29,352,313	29,352,313	+	ENSG00000248391	LINC02064
rs892686		−1	−1			–
rs999354		−1	−1			–
rs1074800		−1	−1			–
rs10781552	10	132,083,729	1.32E + 08	+	ENSG00000188385	JAKMIP3
rs17530232		−1	−1			–
rs1884466		−1	−1			–
rs2300774	3	196,066,841	1.96E + 08	+	ENSG00000072274	TFRC
rs290833		−1	−1			–
rs35161940	17	72,331,083	72,331,083	+	ENSG00000227036	LINC00511
rs4078432		−1	−1			–
rs56970041		−1	−1			–
rs62504403	8	38,946,033	38,946,033	+	ENSG00000169499	PLEKHA2
rs7234058	18	5,830,508	5,830,508	+	ENSG00000261738	MIR3976HG
rs7826267	8	3,097,430	3,097,430	+	ENSG00000183117	CSMD1
rs8003149	14	55,689,786	55,689,786	+	ENSG00000126777	KTN1
rs10404377		−1	−1			–
rs118170811		−1	−1			–
rs12643275		−1	−1			–
rs12904405	15	93,562,186	93,562,186	+	ENSG00000257060	–
rs1999289	21	18,313,675	18,313,675	+	ENSG00000154646	TMPRSS15
rs3098182	15	50,510,783	50,510,783	+	ENSG00000138592, ENSG00000170236	USP8, USP50
rs482905		−1	−1			–
rs6501525	17	72,222,486	72,222,486	+	ENSG00000234899	SOX9-AS1
rs66753613		−1	−1			–
rs6797051		−1	−1			–
rs7076829	10	7,864,783	7,864,783	+	ENSG00000165632	TAF3
rs7514702	1	186,944,891	1.87E + 08	+	ENSG00000116711	PLA2G4A

## 4. Discussion

This study represents a pioneering effort to establish a causal relationship between gut microbiota and ON using a large-scale MR framework. Unlike prior observational studies, which primarily noted associations between dysbiosis and bone health, our MR-based approach identifies specific microbial taxa, such as Erysipelotrichaceae, Christensenellaceae, and Family XIII, as having a causal influence on ON risk. This study’s primary innovation lies in the application of MR to establish causality, overcoming the limitations of observational studies. Furthermore, our identification of 20 host genes associated with ON-specific gut microbial abundance introduces potential mechanistic pathways. These findings align with existing research on the gut–bone axis but further elucidate the direct role of microbial taxa in ON pathogenesis.

While the relationship between the gut microbiota and ON is not fully understood, some studies have indicated that gut dysbiosis may contribute to the development of ON. For instance, Wei et al^[17]^ demonstrated distinct differences in the oral bacterial composition between patients with bisphosphonate-related osteonecrosis of the jaw and individuals without this condition. Mounting evidence suggests that the gastrointestinal tract can communicate with the skeletal system through a mechanism known as the gut-bone axis.^[18]^ Furthermore, a potential etiological link was identified between a decrease in the abundance of the gut bacterium L. animalis and the development of glucocorticoid-induced osteonecrosis of the femoral head.^[19]^ Research has shown a connection between rheumatoid arthritis and changes in gut microbiota and short-chain fatty acids levels. By adjusting the composition of gut microbiota to restore levels of short-chain fatty acids, it is possible to uphold gut barrier integrity, support immune homeostasis, reduce inflammatory responses associated with rheumatoid arthritis, and prevent bone loss.^[20]^ These findings highlight the potential involvement of the gut microbiota in the pathogenesis of ON and suggest potential therapeutic interventions. For example, research demonstrated that glucocorticoids can reduce levels of intestinal Lactobacillus animalis, and that oral supplementation with Lactobacillus animalis attenuates glucocorticoid-induced osteonecrosis of the femoral head by promoting angiogenesis, enhancing osteogenesis, and decreasing apoptosis.^[19]^

Accumulated research has demonstrated the microbiome’s significant role in maintaining the health and function of extraintestinal organs.^[21]^ Disruption of microbiota homeostasis results in heightened translocation of the microbiota and its bacterial byproducts, such as lipopolysaccharides and short-chain fatty acids, which subsequently trigger systemic and local immune responses.^[22]^ This phenomenon allows the gut microbiota to exert influence on distant organs, including the skeletal system.^[23]^ Interactions between the gut microbiota and the host impact bone mass equilibrium, with studies showing increases in species such as Faecalibacterium, Fusobacterium, and Tolumonas, and decreases in Roseburia spp. and Bacteroides in osteoporosis.^[21]^ Additionally, the gut microbiota may influence bone health by modulating levels of steroid hormones, insulin-like growth factor 1, parathyroid hormone, and vitamin D metabolites, affecting osteoblasts (for bone formation) and osteoclasts (for bone resorption).^[21]^ Short-chain fatty acids produced by gut microbiota contribute to antioxidant production, which helps mitigate oxidative stress, offering insights into the pathogenesis of ON. Further studies are needed to validate these causal relationships and explore their potential for guiding prevention and treatment strategies.^[24]^

Our findings indicate that 3 gut microbiota taxaorder Erysipelotrichales, family Erysipelotrichaceae, and class Erysipelotrichia are positively correlated with the risk of ON. This represents the first reported association between these taxa and ON based on large sample data. The potential role of family Erysipelotrichaceae in human health and disease is gaining attention, with variations observed in populations with smoking populations, colorectal cancer patients, and individuals with inflammatory bowel disease.^[25]^ Erysipelotrichaceae is associated with lipid metabolism, systemic inflammation, and cholesterol metabolism-related products.^[26]^ Given that lipid and cholesterol metabolism disorders elevate the risk of osteonecrosis of the femoral head,^[27]^ and that chronic inflammation is a significant risk factor for ON,^[28]^ these associations merit further study. Erysipelotrichales is also recognized as a source of butyric acid, a gut-protective short-chain fatty acid in athletes.^[29]^ Butyrate has been shown to promote osteogenic differentiation of stromal cells, mineralized nodule formation, and bone resorption via osteoclast activity.^[23]^ This effect may involve immunostimulation and pro-osteoclastogenic cytokines increase.^[30]^ Elevated butyrate levels also increase Treg cells, which activates Wnt signaling in osteoblasts to stimulate bone formation.^[31]^ Additionally, butyrate inhibits the differentiation of primary bone marrow cells into osteoclasts.^[30]^ Erysipelotrichaceae has been documented to support bone formation, potentially enhanced by diallyl trisulfide to prevent age-related bone degeneration.^[32]^ Concurrently, research revealed a reduction in the abundance of Erysipelotrichaceae during episodes of acute gouty arthritis, and modified Baihu decoction may alleviate the disease by altering the gut microbiota to control the immune-inflammatory response.^[33]^ Previous research has established a robust correlation between alterations in gut microbiota and both bone mass and bone metabolism. A study found that infrared supplementation could impact bone metabolism beneficially in rats by influencing the volume of certain gut microbiota, including Erysipelotrichaceae.^[34]^ Specifically, Christensenellaceae may exert protective effects by promoting metabolic homeostasis and reducing inflammatory cytokine levels, which supports bone formation.^[35]^ Although there are limited studies on Erysipelotrichales and ON, the existing literature strongly suggests a potential link between them, offering new perspectives for future research.

The Christensenellaceae family, first identified in 16S rRNA gene sequences from healthy Japanese males, belongs to the phylum Firmicutes.^[36]^ Studies show that Christensenellaceae abundance is correlated with race, sex, and heritability.^[35]^ It is inversely associated with body mass index and linked to lipid metabolites, including triglycerides, high-density lipoprotein, total cholesterol, low-density lipoprotein, and apolipoprotein B.^[35]^ Lipid metabolism abnormalities are present in both alcoholic and hormonal osteonecrosis.^[5]^ In glucocorticoid-induced osteoporosis in rats, Christensenellaceae_R_7_group abundance increased significantly, with Lactobacillus plantarum alleviating the disease by modulating intestinal flora.^[37]^ While Christensenellaceae has not previously been associated with ON, our findings reveal a negative association, suggesting new avenues for further research.

Due to the paucity of studies on FamilyXIII, we found no further research on its association with ON. Only a few studies showed changes in Family XIII abundance in the gut microbiota of patients with dry syndrome and negative cognitive processing bias.^[38]^ One study demonstrated significant differences in the composition of the gut microbiota, including the presence of Family_XIII and Erysipelotrichaceae, between healthy rats and rats with rheumatoid arthritis, and Ershiwuwei Lvxue Pill could modulate the abundance of these intestinal flora to alter osteoclast activity to alleviate the symptoms of rheumatoid arthritis.^[39]^ Therefore, our results indicate, for the first time, a negative association between FamilyXIII and the risk of ON development, which may inspire future studies on FamilyXIII and ON.

Research has shown that gut microbes influence gene expression, regulate host physiology, and may contribute to disease.^[40]^ Recent studies have indicated that gut microbes induce cytokines to regulate bone metabolism and down-regulate gut-derived serotonin, impacting bone metabolism.^[41]^ These findings underscore the significant role of gut microbe-host gene interactions in bone metabolism-related diseases. Changes in gut microbiota have been observed in nontraumatic osteonecrosis of the femoral head induced by steroids and alcohol. Specifically, Lactobacillus and Roseburia levels increased in alcohol-induced ON, while Megasphaera and Akkermansia were prevalent in steroid-induced ON.^[42]^ In medication-related osteonecrosis of the jaw, inflammation, infection, and immune dysfunction, suggest a potential gut microbiota association, with elevated levels of Actinobacillus spp. and Prevotella in the necrotic bone region.^[19]^ Through SNP annotation, we identified 20 host genes potentially associated with ON-specific gut microbial abundance, such as USP8 (ubiquitin-specific protease 8) and TFRC (encoding Tfr1, transferrin receptor 1). In experimental studies in mice, USP8 was found to regulate Wnt/β-catenin signaling in osteoblasts, crucial for osteogenic differentiation of skeletal progenitors.^[43]^ Additionally, disruption of TFRC-encoded Tfr1 expression was observed to attenuate mitochondrial metabolism and cytoskeletal organization of osteoblasts in vitro, resulting in reduced bone resorption.^[44]^ These observations lead us to speculate that these genes may be related to the mechanistic occurrence of ON.

Research indicates that Lactobacillus plantarum mitigates glucocorticoid-induced osteoporosis by modulating rat gut microbiota composition, notably increasing the abundance of Christensenellaceae_R. Additionally, it alters serum metabolic profiles, leading to a significant increase in serum pyrazine and γ-glutamylcysteine levels. These changes collectively inhibit osteoclast formation and promoting osteoblast formation.^[37]^ These findings offer valuable perspectives in ON research, where osteoporosis and changes in cellular inflammatory markers are common across various forms of ON including alcohol-induced, drug-related, and glucocorticoid-induced ON.^[2]^ Prior studies support that shifts in gut bacterial populations can influence bone metabolism through inflammatory pathways. For instance, unclassified Desulfovibrionaceae have been positively associated with the upregulation of cellular inflammatory markers, such as tumor necrosis factor-α, interleukin-6, interleukin-1β, and monocyte chemoattractant protein-1.^[37]^ An upsurge in these pro-inflammatory cytokines may impact osteoblasts or osteoclasts directly or indirectly, facilitating the differentiation of monocytes to mature osteoclasts, thus exacerbating bone resorption and reducing bone mass. The above studies have established potential associations between various forms and sites of ON and the gut microbiota. However, the FinnGen Project Database does not currently distinguish among specific ON types or sites, which limits the broad applicability of these findings. Thus, closely tracking updates is essential to refine and enhance the quality of the research.

The study broadens the scope of osteoimmunology and osteomicrobiology by demonstrating how specific gut microbiota taxa influence skeletal conditions. These findings pave the way for microbiota-targeted therapies, emphasizing their potential in precision medicine. However, our study has limitations. One limitation of this study is the use of GWAS datasets derived primarily from individuals of European ancestry. This may restrict the generalizability of our findings to other populations, particularly those with differing genetic backgrounds and gut microbiota compositions. Evidence suggests that gut microbiota diversity varies significantly across populations due to dietary, environmental, and genetic factors.^[45]^ Future research should validate these findings across diverse populations, explore specific ON etiologies and anatomical sites, evaluate microbiota-targeted interventions like Christensenellaceae supplementation, and utilize multi-omics approaches to uncover detailed gut microbiota-host interactions. The occurrence of bisphosphonate-associated osteonecrosis correlates with dosage, treatment duration, administration method, and the presence of cofactors including glucocorticoid and immunosuppressant usage, as well as dental interventions. Further research is need to determine whether these factors also affect gut microbiota alterations.^[46]^ Studies indicated that gender-affirming hormone therapy promotes skeletal maturation in young male mice via the gut microbiome, but not in females, suggesting that gender may influence gut microbiome differentiation.^[47]^ Additionally, distinct shifts in the gut microbiota were identified in rat models of postmenopausal, ovariectomy-induced, and glucocorticoid-induced osteoporosis, highlighting a complex relationship between bone health and gut microbial composition.^[48]^ Therefore, we aim to integrate the latest data, analyzing disease-related factors like age, medication, gender, and other potentially relevant factors in subgroups to enhance the study robustness.

## 5. Conclusions

This Mendelian Randomization study identifies a causal relationship between specific gut microbiota taxa and osteonecrosis (ON), with Erysipelotrichales, Erysipelotrichaceae, and Erysipelotrichia increasing ON risk, while Christensenellaceae R and Family XIII are protective. Mechanisms may involve lipid metabolism, immune modulation, and osteogenic differentiation, mediated by genes such as USP8 and TFRC. These findings introduce novel insights into the gut-bone axis, emphasizing gut microbiota’s role in ON pathogenesis and identifying host genes that may mediate these effects. Future research should validate these results in diverse populations, explore mechanistic pathways using multi-omics approaches, and conduct interventional studies to assess therapeutic efficacy, paving the way for improved ON outcomes.

## Acknowledgments

We would like to thank Dr. Fan Zhenliang (Zhejiang Provincial Hospital of Traditional Chinese Medicine) for his assistance with the statistical analysis of the data in this article.

## Author contributions

**Conceptualization:** Yong Cai, Chaoqing Zhou, Jun Zhao.

**Data curation:** Chaoqing Zhou, Jun Zhao.

**Formal analysis:** Chaoqing Zhou, Jun Zhao.

**Investigation:** Junjie Guan, Jizhao Jiang, Jun Zhao.

**Methodology:** Junjie Guan, Jizhao Jiang.

**Resources:** Jizhao Jiang, Xingshi Zhang.

**Software:** Junjie Guan.

**Supervision:** Bo Dai, Jizhao Jiang, Jun Zhao.

**Validation:** Bo Dai, Xingshi Zhang.

**Visualization:** Bo Dai, Xingshi Zhang.

**Writing – original draft:** Yong Cai, Jun Zhao.

**Writing – review & editing:** Yong Cai, Jun Zhao.

## Supplementary Material
