# Supplementary material for: Gut microbiota and osteonecrosis: A Mendelian randomization study
Source: Medicine (Baltimore). 2025 Mar 7;104(10):e41703. doi: 10.1097/MD.0000000000041703 (PMC11902940; doi:10.1097/MD.0000000000041703)
Supplement: Supplementary file 2 [file medi-104-e41703-s002.pdf]

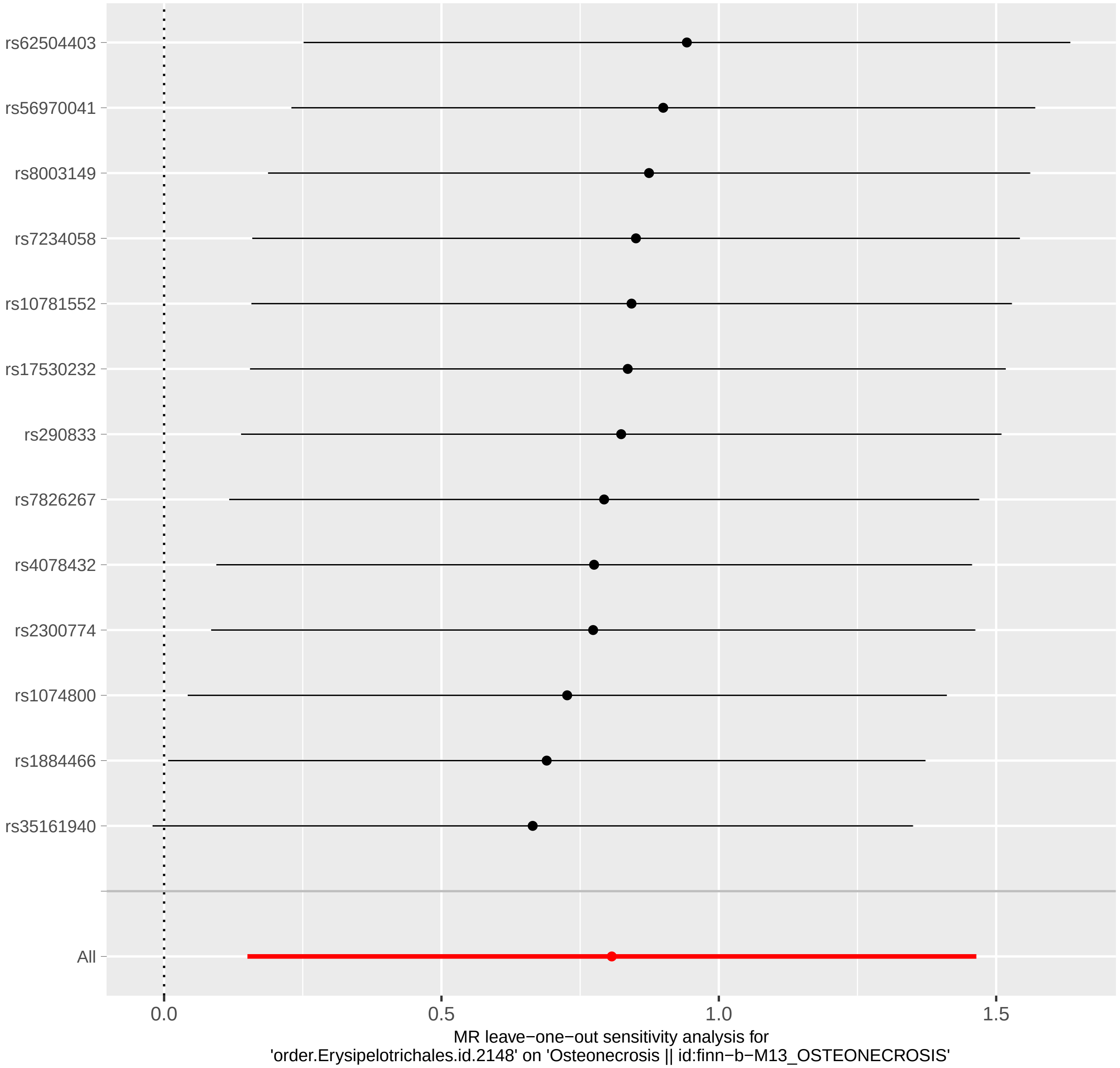

rs62504403
rs56970041
rs8003149
rs7234058
rs10781552
rs17530232
rs290833
rs7826267
rs4078432
rs2300774
rs1074800
rs1884466
rs35161940
All
0.0
0.5
1.0
1.5
MR leave−one−out sensitivity analysis for
'order.Erysipelotrichales.id.2148' on 'Osteonecrosis || id:finn−b−M13_OSTEONECROSIS'

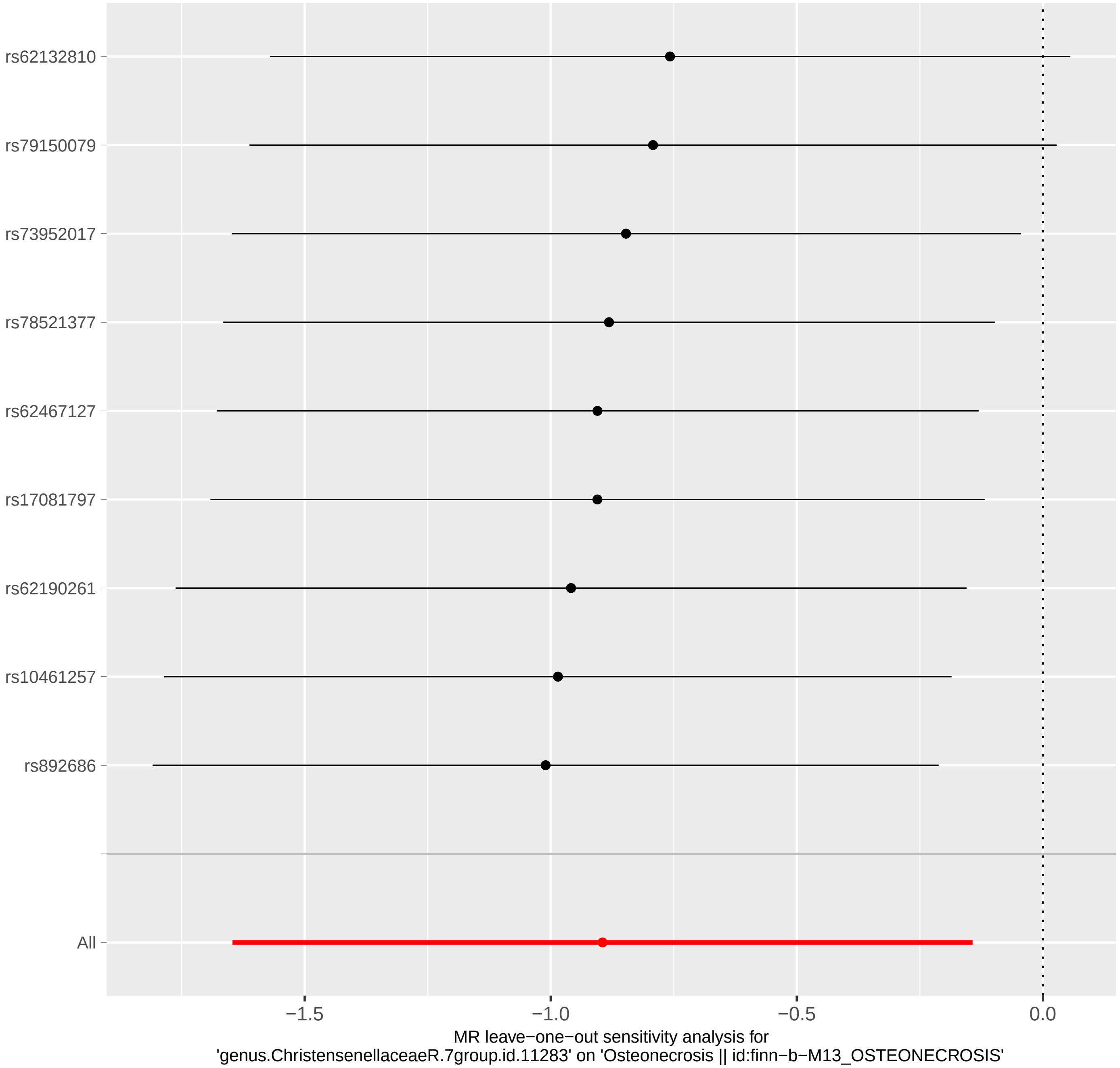
rs62132810
rs79150079
rs73952017
rs78521377
rs62467127
rs17081797
rs62190261
rs10461257
rs892686
All
−1.5
−1.0
−0.5
0.0
MR leave−one−out sensitivity analysis for
'genus.ChristensenellaceaeR.7group.id.11283' on 'Osteonecrosis || id:finn−b−M13_OSTEONECROSIS'

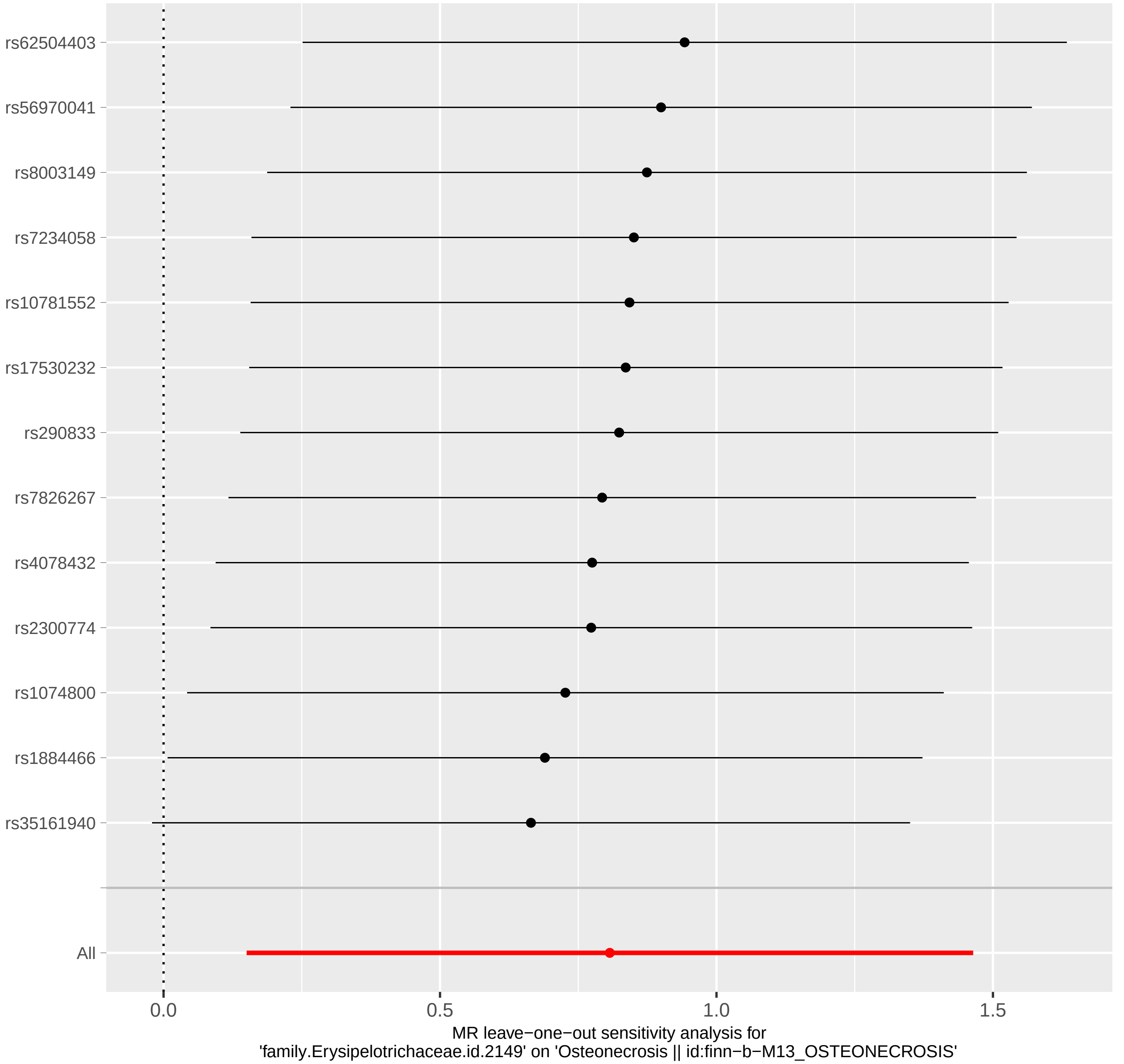
rs62504403
rs56970041
rs8003149
rs7234058
rs10781552
rs17530232
rs290833
rs7826267
rs4078432
rs2300774
rs1074800
rs1884466
rs35161940
All
0.0
0.5
1.0
1.5
MR leave−one−out sensitivity analysis for
'family.Erysipelotrichaceae.id.2149' on 'Osteonecrosis || id:finn−b−M13_OSTEONECROSIS'

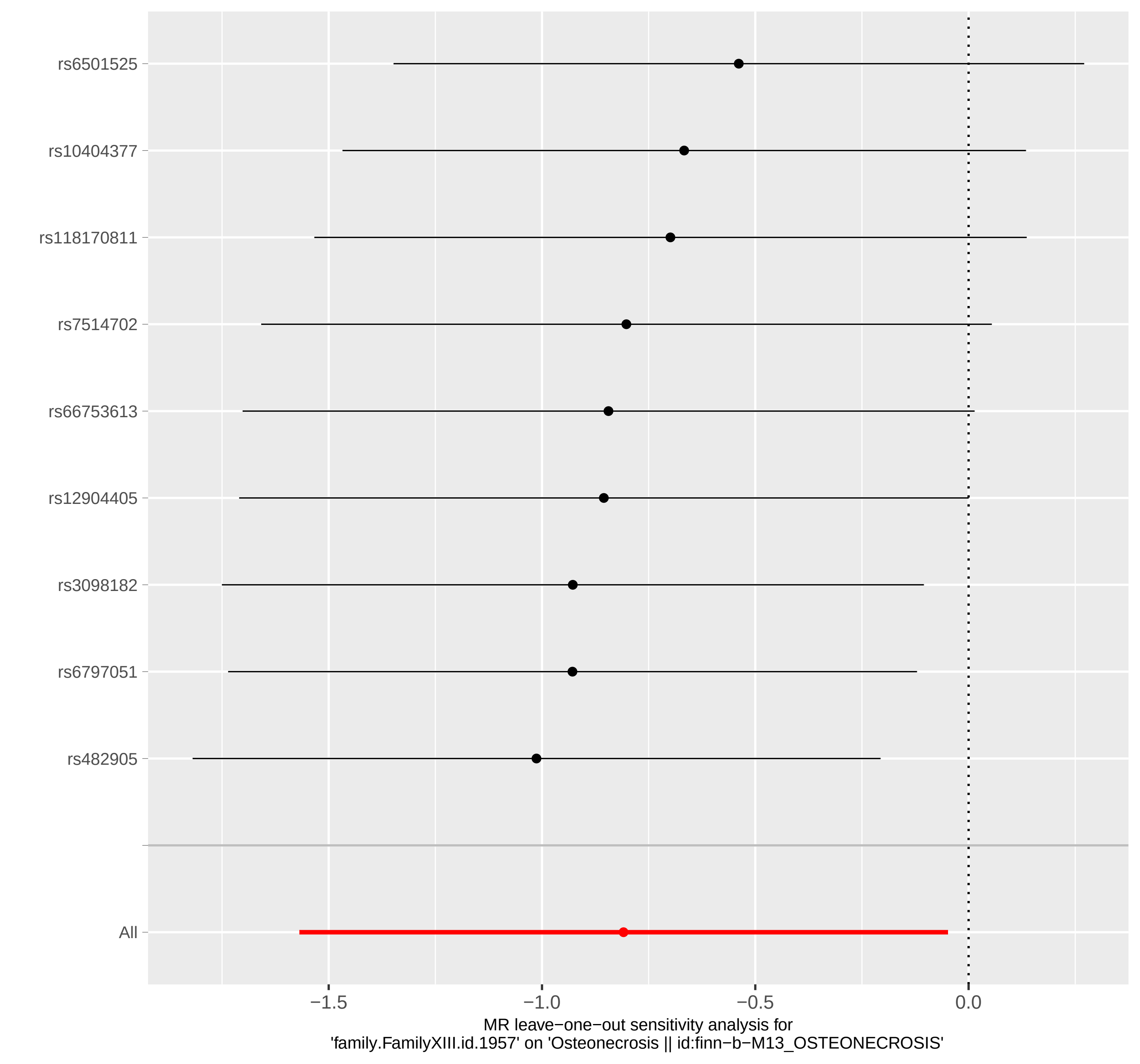
rs6501525
rs10404377
rs118170811
rs7514702
rs66753613
rs12904405
rs3098182
rs6797051
rs482905
All
−1.5
−1.0
−0.5
0.0
MR leave−one−out sensitivity analysis for
'family.FamilyXIII.id.1957' on 'Osteonecrosis || id:finn−b−M13_OSTEONECROSIS'

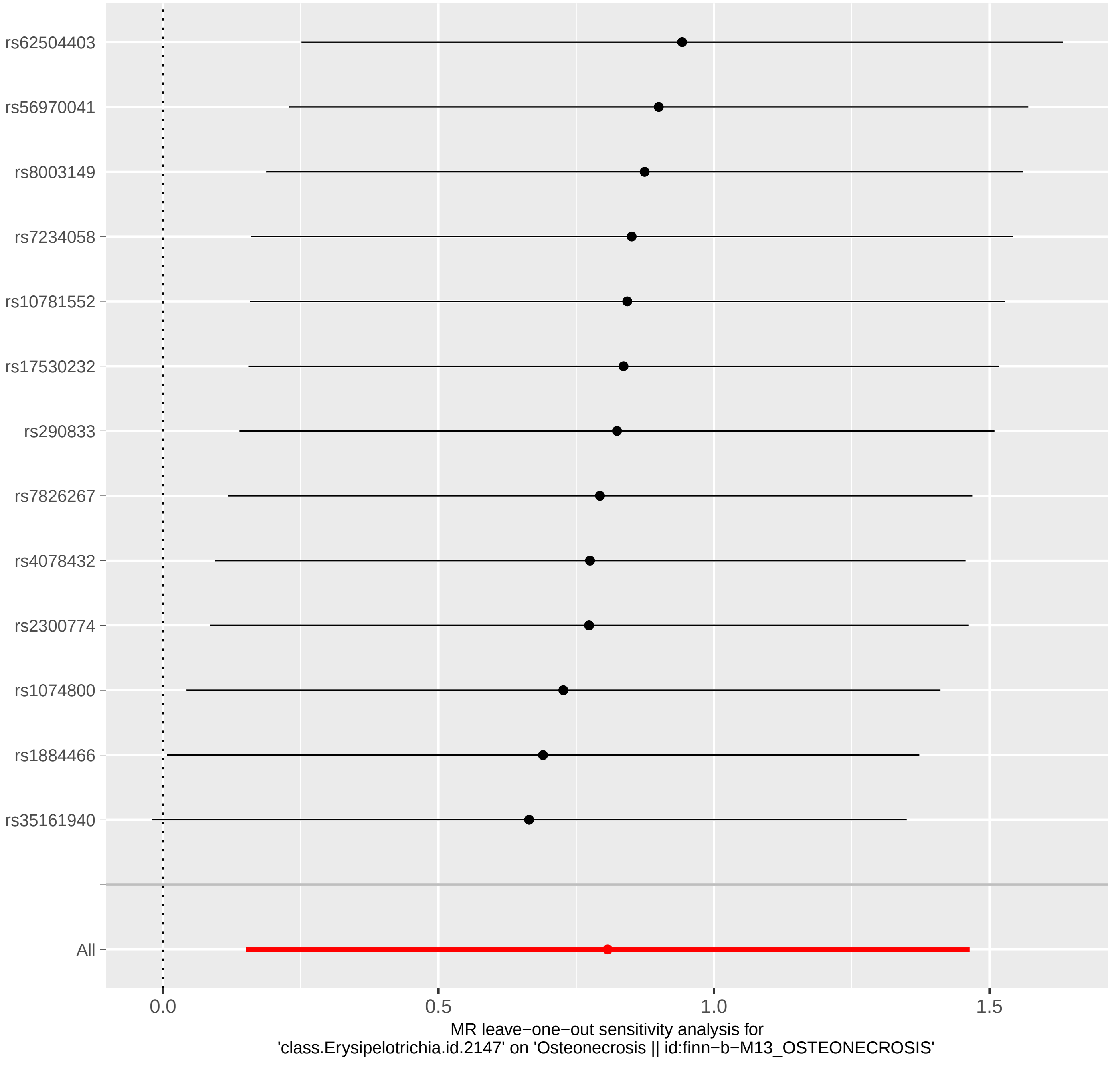

rs62504403
rs56970041
rs8003149
rs7234058
rs10781552
rs17530232
rs290833
rs7826267
rs4078432
rs2300774
rs1074800
rs1884466
rs35161940
All
0.0
0.5
1.0
1.5
MR leave−one−out sensitivity analysis for
'class.Erysipelotrichia.id.2147' on 'Osteonecrosis || id:finn−b−M13_OSTEONECROSIS'
